# Eco-friendly silver nanoparticle-based dual sensors for environmental toxicants: Hg^2+^ and H_2_O_2_

**DOI:** 10.1039/d6ra02784a

**Published:** 2026-07-16

**Authors:** Md. Ahad Mahamud Nahim, Md Toufiqul Islam, Saurav Kumar Das, A. B. M. Nazmul Islam, Rumpa Kundu, Md. Abu Rayhan Khan, Shofiur Rahman, Mahmoud A. Gawati, Habib Md. Ahsan

**Affiliations:** a Chemistry Discipline, Khulna University 3rd Academic Building Khulna-9208 Bangladesh ahsanhru@chem.ku.ac.bd; b Graduate School of Environmental Studies, Tohoku University 6-6-20 Aoba Aramaki, Aoba-ku Sendai 980-8579 Japan; c Department of Chemistry, Mississippi State University USA; d Biological and Environmental Sensing Research Unit, King Abdullah Institute for Nanotechnology, King Saud University Riyadh 11451 Saudi Arabia mrahman1@ksu.edu.sa

## Abstract

Monitoring mercury ions (Hg^2+^) and hydrogen peroxide (H_2_O_2_) in aquatic environments is essential due to their environmental persistence and ability to induce severe health disorders. This study highlights the potential of biogenically synthesized silver nanoparticles (AgNPs) as an efficient probe for Hg^2+^ and H_2_O_2_ detection in water. The AgNP nanoprobes are highly selective for Hg^2+^, as evidenced by the surface plasmon resonance (SPR) band and a visible color shift from brown to colorless; interference studies confirmed minimal responses to competing ions. The nanoprobe exhibited a limit of detection (LOD) of 1.65 µM and a limit of quantification (LOQ) of 5.50 µM, with excellent linearity (*R*^2^ = 0.9919). The nanoprobe was tested for Hg^2+^ in real water samples and showed excellent recovery rates. Analysis using synchrotron radiation X-ray photoelectron spectroscopy (SR-XPS) showed that the formation of an amalgam during detection alters the SPR. Additionally, the AgNP probe efficiently detected hydrogen peroxide (H_2_O_2_) in aqueous solution, with an LOD of 5.46 µM and an LOQ of 18.20 µM, respectively. The study emphasizes that a rapid, eco-friendly colorimetric sensor was developed to reliably detect hazardous pollutants in water.

## Abstract 2

An AgNP-based colorimetric sensor for selective Hg^2+^ and H_2_O_2_ detection. The mechanism leverages Ag–Hg amalgam formation, enabling rapid, “naked-eye” monitoring of environmental and biochemical contaminants in aqueous samples.

## Graphical abstract

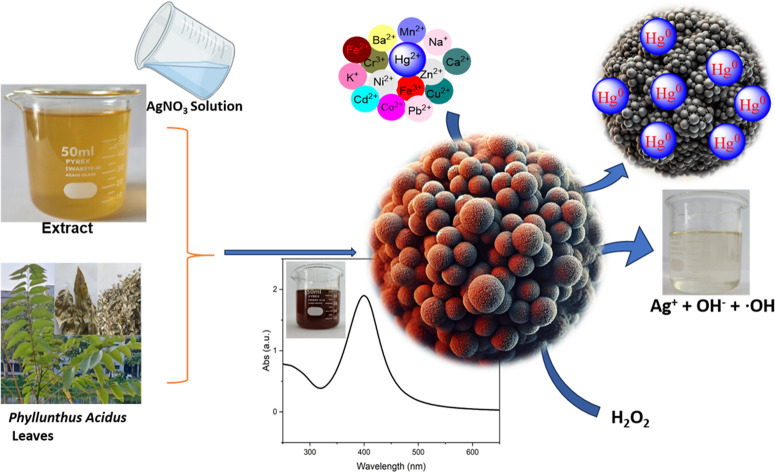

## Introduction

Mercury is a highly persistent neurotoxicant that poses a severe threat to both ecological stability and human health. While natural phenomena such as volcanic activity and crustal degassing contribute to its presence, anthropogenic activities, including industrial discharges and coal combustion, remain the primary drivers of mercury pollution.^1^ Once released, mercury undergoes bioaccumulation through the food chain, where even trace exposure can lead to debilitating disorders of the central nervous system, kidneys, and endocrine functions.^2–4^ Similarly, hydrogen peroxide, though essential as a biological signaling molecule, is a potent oxidant that at elevated concentrations causes significant respiratory and gastrointestinal damage.^5,6^ Given the acute toxicity of these pollutants, the development of sensing platforms that are both highly sensitive and capable of rapid, on-site deployment is a global environmental priority. Analytical techniques that can reliably detect mercury ions in the environment include atomic absorption/emission spectrometry (AAS/AES),^7^ chemiluminescent (CL) immune chromatographic assay strip (ICAS),^8^ surface-enhanced Raman scattering (SERS),^9^ high-performance liquid chromatography (HPLC),^10^ electrochemical sensors,^11^ and inductively coupled plasma atomic emission spectroscopy (ICP-AES),^12^ offer excellent sensitivity and low detection limits for Hg^2+^ detection. However, these methods are often hampered by high operational costs, the need for sophisticated instrumentation, and complex sample preparation protocols. Crucially, these laboratory-bound techniques are unsuitable for real-time, field-level monitoring in remote areas. Consequently, there is an urgent demand for “point-of-care” environmental sensors that are cost-effective, portable, and simple to operate without compromising precision. Nanotechnology is expected to play a pivotal role in advancing the development of nanoprobes for the detection of environmental pollutants.^13^ Among various nanomaterials, AgNPs are particularly distinguished by their exceptional SPR properties, which yield high extinction coefficients and distinct optical signatures.^14,15^ Colorimetric sensing using metal nanoparticles has become one of the most appealing methods because it is simple, quick, and easy to see due to their strong SPR properties, which are highly sensitive to changes in particle size, shape, and interparticle spacing.^16,17^ To date, various AgNP-based colorimetric strategies have been developed for Hg^2+^ detection, primarily relying on mechanisms such as ligand-receptor-induced nanoparticle aggregation, dithiothreitol-mediated assemblies, or the oxidative dissolution of AgNPs driven by the high redox potential of Hg^2+^/Hg (0.85 V *vs.* NHE).^18,19^ While these methods offer impressive sensitivity, they often suffer from poor selectivity against other heavy metals, require complex surface functionalization with synthetic thiols or DNA aptamers, or lack a deep structural validation of the resulting surface states.^20^ Upon interaction with target analytes, such as Hg^2+^, unfunctionalized biogenic nanoparticles undergo aggregation or morphological changes, resulting in a visible color shift and a corresponding alteration in the SPR absorption band, which can be visualized by the naked eye even at low concentrations.^21,22^ Furthermore, the unique ability of mercury to form amalgams with other metals significantly influences the optical and electronic properties of metal nanoparticles.^23,24^ This amalgamation process shifts the SPR band and triggers a visible color transition, providing a rapid and quantifiable readout. Similarly, the catalytic or oxidative interaction between AgNPs and H_2_O_2_ enables sensitive detection *via* predictable spectral changes. While conventional AgNPs are effective, their synthesis often involves hazardous chemical reductants which limits their “green” credentials. This study addresses this gap by utilizing *Phyllanthus acidus* (locally known as “Orboroi” in Bangladesh) as a sustainable, biogenic source for AgNP fabrication. The leaf extract is rich in bioactive phytochemicals, including flavonoids, tannins, and phenolics, which serve as dual reducing and capping agents, eliminating the need for secondary synthetic surface functionalization.^25^ This approach not only leverages the regional biodiversity of the Khulna and Chittagong divisions but also ensures that the resulting sensor is eco-friendly from production to application. By integrating SR-XPS characterization to understand the detection process with real-world water analysis, this work provides a comprehensive framework for the dual-mode detection of Hg^2+^ and H_2_O_2_, offering a robust and scalable solution for monitoring hazardous pollutants in aquatic ecosystems.

## Results and discussion

**Scheme 1:**
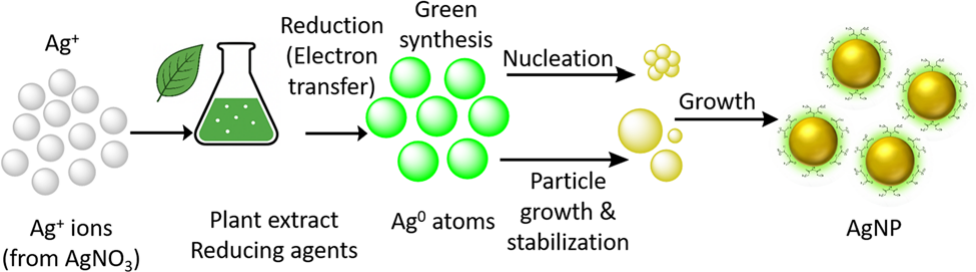
Schematic representation AgNPs formation.

The synthesis of AgNPs involves a multi-step process: the reduction of silver ions (Ag^+^) to metallic silver atoms (Ag^0^), followed by nucleation and subsequent growth into stable nanostructures (Scheme 1).^26^ In this study, the aqueous extract of *Phyllanthus acidus* functioned as a dual-action agent, providing both the reducing power and the stabilizing (capping) matrix. Phytochemical screening of the *Phyllanthus acidus* extract confirmed a rich profile of bioactive constituents, including alkaloids, flavonoids, phenolic acids, and vitamins. These metabolites, particularly the polyphenols and flavonoids, are instrumental in transferring electrons to Ag^+^ ions,^27^ while their bulky molecular structures provide steric stabilization, preventing nanoparticle indentation or aggregation.

**Fig. 1:**
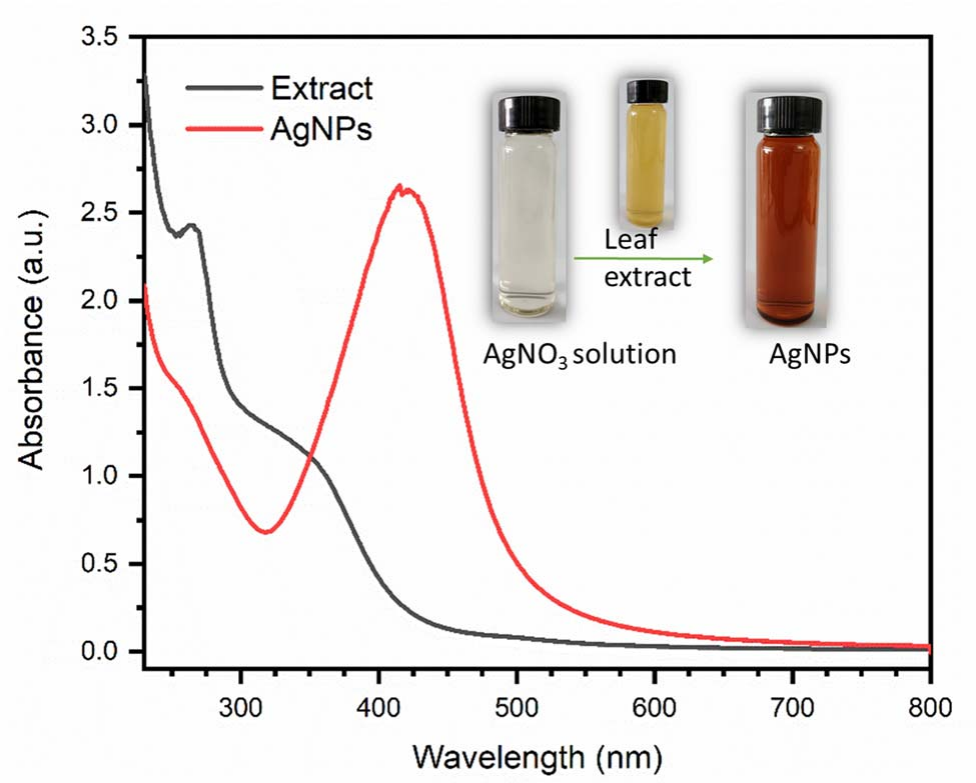
UV-visible spectra of AgNPs and *Phyllanthus acidus* leaf extract. Inside the color of AgNO_3_ solution, represent the highly concentrated, undiluted state post-synthesis, leaf extract and AgNPs. Synthesis condition: 1 mL of extract, 50 mL of 1 mM AgNO_3_, 60 °C, 15 minutes, and pH 10.

The successful formation of AgNPs was primarily monitored *via* UV-vis spectroscopy, as silver nanoparticles exhibit a characteristic SPR due to the collective oscillation of conduction electrons. Upon mixing the *Phyllanthus acidus* extract with the AgNO_3_ solution, a rapid color transition from pale yellow to a deep brownish-red was observed within 15 minutes at 60 °C. This visual change was corroborated by a sharp, prominent SPR absorption band centered at 418 nm (Fig. 1).

The symmetry and intensity of this peak suggest the formation of relatively monodisperse, spherical nanoparticles. The absence of secondary peaks at higher wavelengths further indicates that biogenic synthesis successfully produced discrete particles without significant agglomeration.^28^

To ensure the production of stable and reproducible AgNPs for sensing applications, we systematically optimized several experimental variables: temperature, extract volume, AgNO_3_ concentration, reaction time, and pH. The thermal energy of the reaction system significantly influenced the reduction kinetics of Ag^+^ ions. At room temperature (25 °C), AgNP formation was initiated, but the low kinetic energy of the*Phyllanthus acidus* reducing agents resulted in a slow nucleation rate. As the temperature increased, the SPR band intensity rose, reflecting an accelerated reduction process (Fig. S1). However, at 70 °C, the absorbance decreased, which is characteristic of Ostwald ripening or the formation of larger, non-uniform clusters. The optimal balance between rapid nucleation and controlled growth was achieved at 60 °C. Time-resolved UV-vis spectra (Fig. S2) demonstrated a progressive increase in SPR intensity, reaching equilibrium at 15 minutes. The cessation of absorbance growth after this threshold confirms the total consumption of available Ag^+^ ions. The stoichiometry between the precursor and the reductant was equally critical. While 1 mL of*Phyllanthus acidus* extract provided the ideal concentration of capping agents to maintain stability, higher volumes led to a decline in SPR intensity, likely due to secondary reduction processes or destabilization of the capping layer (Fig. S3). Similarly, 1 mM AgNO_3_ was identified as the optimal precursor concentration. Higher concentrations resulted in a noticeable red shift (Fig. S4), indicating a transition from spherical nanoparticles to larger aggregates due to an oversupply of silver ions relative to the stabilizing capacity of the extract. The pH of the reaction medium played a decisive role in the reduction potential of the phytochemicals. At pH 10, the synthesis reached its maximum efficiency, evidenced by the highest SPR absorbance (Fig. S5). This is attributed to the deprotonation of phenolic and flavonoid groups within the extract at alkaline levels. This deprotonation increases the electron-donating capability of these biomolecules, thereby facilitating a more rapid and complete reduction of Ag^+^ to Ag^0^.^29^ The synergistic optimization of these parameters led to the following protocol for the production of highly stable, monodisperse AgNPs using 50 mL of 1 mM AgNO_3_ and 1 mL of *Phyllanthus acidus* extract, maintained at 60 °C for 15 minutes, at pH 10. This standardized approach ensures a consistent, high-intensity SPR profile, providing a reliable foundation for sensitive colorimetric detection.

**Fig. 2:**
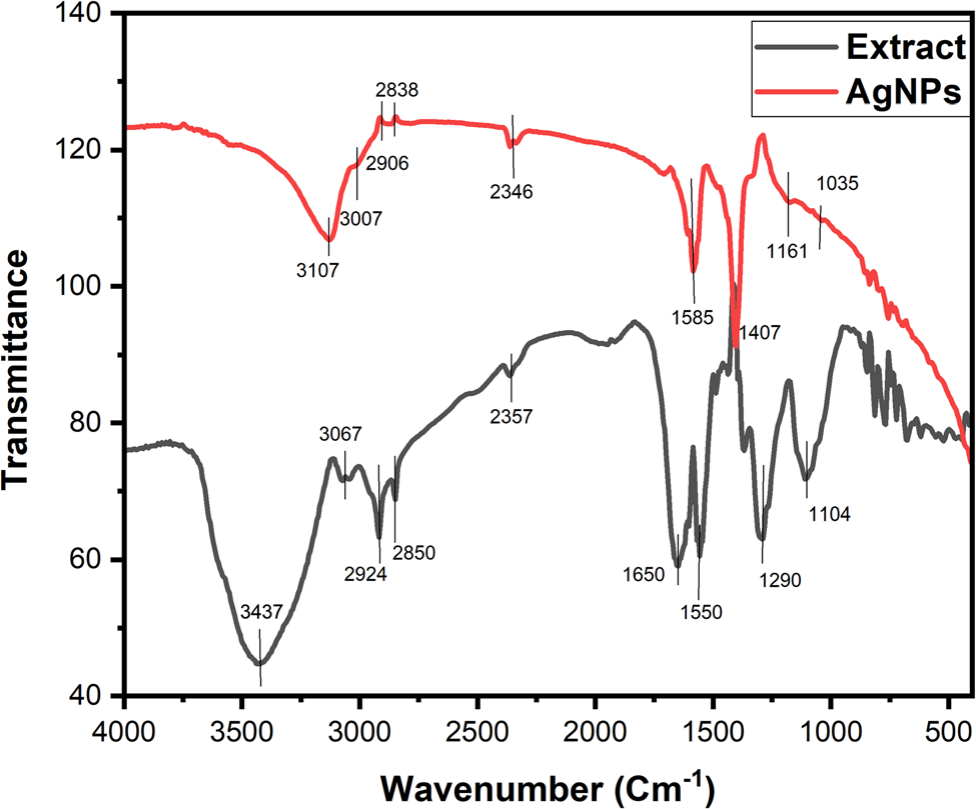
FTIR spectra of *Phyllanthus acidus* leaf extract and AgNPs synthesized using *Phyllanthus acidus* leaf extract. (Condition: 1 mL of extract, 50 mL of 1 mM AgNO_3_, 60 °C, 15 minutes, and pH 10).

FTIR spectroscopy was employed to identify the phytochemical functional groups in the *Phyllanthus acidus* extract and to elucidate their role in the reduction and stabilization of AgNPs (Fig. 2).

FTIR spectroscopy was employed to identify the phytochemical functional groups in the *Phyllanthus acidus* extract and to elucidate their role in the reduction and stabilization of AgNPs (Fig. 2).

The leaves extract exhibits a complex spectral profile consistent with its rich composition of bioactive metabolites, including terpenoids, flavonoids, and phenolics.^25^ A prominent, broad absorption band at 3437 cm^−1^ is attributed to the O–H stretching vibrations of polyphenolic compounds. The presence of long-chain hydrocarbons, typical of terpenoids, is confirmed by a C–H stretching vibration at 2920 cm^−1^. Furthermore, a strong signal at 1650 cm^−1^ indicates the presence of carbonyl (C=O) groups, while the peak at 1550 cm^−1^ corresponds to aromatic C=C and C=N stretching, suggesting the presence of purine-ring derivatives and polyphenols.^28^ Upon the formation of capped AgNPs, significant spectral shifts and intensity changes were observed, providing clear evidence of the biogenic reduction process. The broad hydroxyl peak shifted from 3437 to 3107 cm^−1^, indicating the active involvement of polyphenolic groups in the reduction of Ag^+^ and subsequent hydrogen bonding during capping. Subtle shifts in peaks at 1585, 1407, and 1161 cm^−1^ (relative to the crude extract) suggest that carbonyl and amine groups are instrumental in coordinating with the nanoparticle surface. These results confirm that the phytochemicals from *Phyllanthus acidus* act as a multi-functional matrix, where polyphenolic compounds reduce Ag^+^ ions to Ag^0^, while other secondary metabolites provide a protective capping layer that ensures long-term colloidal stability.

The stability of the biogenically synthesized AgNPs was evaluated by measuring their Zeta potential (*ζ*-potential), which provides a quantitative measure of the surface charge and the magnitude of electrostatic repulsion between particles in a colloidal suspension. According to DLVO theory, the *ζ*-potential is a critical indicator of long-term stability, as a higher net surface charge generates sufficient repulsive forces to counteract the attractive van der Waals interactions that lead to aggregation. As illustrated in Fig. S6, the synthesized AgNPs exhibited a significant negative *ζ*-potential of −23.53 mV. This substantial negative value confirms the presence of a robust surface charge, primarily attributed to the capping action of bioactive constituents from the *Phyllanthus acidus* extract. During the synthesis process, phytochemicals, particularly those containing oxygen-rich functional groups like hydroxyl (–OH) and carboxyl (–COO^−^) adsorb onto the nanoparticle surface. The deprotonation of these groups (consistent with the optimized pH 10 conditions) imparts a dense negative charge to the AgNPs.^29^ The resulting strong electrostatic repulsion effectively prevents nanoparticle clustering, ensuring a stable and uniform dispersion within the aqueous medium. Consequently, the *ζ*-potential analysis validates that the using *Phyllanthus acidus* -mediated AgNPs possess the necessary colloidal integrity for reliable sensing applications. Furthermore, we monitored the long-term storage stability of the synthesized AgNPs stored at 4 °C over 9 months. The stability was evaluated by recording the UV-vis absorption spectra at regular intervals to check for changes in the Surface Plasmon Resonance (SPR) peak, and shown in SI Fig. S7. The results show that the characteristic SPR peak remained tightly centered at 418, with no significant decrease in absorbance intensity or broadening of the peak. This indicates that the biomolecules in the *Phyllanthus acidus* leaf extract effectively act as capping agents, preventing the aggregation of AgNPs over extended storage. To quantify the organic fraction and verify the presence of the capping layer on the nanoparticle surface, thermogravimetric analysis (TGA) was performed. As illustrated in the TGA thermogram (Fig. S8), the AgNPs exhibited a distinct, two-stage thermal degradation profile. Following an initial minor weight loss attributed to the evaporation of chemisorbed water, a sharp and progressive 13% weight loss was observed between 212 °C and 380 °C.^30^ This significant mass reduction is directly linked to the thermal decomposition and volatilization of the bio-organic moieties, specifically the phytochemicals and heat-resistant aromatic compounds, that constitute the capping shell. The stability of the weight beyond 400 °C represents the remaining metallic silver core. These results confirm that the *Phyllanthus acidus* extract does not merely reduce the silver ions but successfully anchors a robust organic layer onto the AgNP surface. This bio-organic coating is essential for maintaining the interparticle distance and preventing irreversible aggregation, further validating the long-term colloidal stability of the sensor.

The elemental composition and purity of the biogenic AgNPs were determined through Energy Dispersive X-ray (EDX) analysis. The resulting spectrum (Fig. S9) confirms a high metallic content, with silver (Ag) accounting for 85.23% of the total elemental weight.^31^ The appearance of sharp, high-intensity peaks characteristic of silver validates the successful reduction and nucleation of the Ag^+^ precursor into a concentrated metallic phase. In addition to the primary silver signals, the analysis detected significant quantities of carbon (5.43% C), oxygen (8.40% O), and nitrogen (0.94% N). These elements are derived from the biomolecular backbone of the *Phyllanthus acidus* leaf extract. Their presence in the EDX spectrum provides direct evidence of the organic “capping” layer that encapsulates the silver core. These phytochemical barriers, composed of flavonoids, phenolics, and proteins, impart the necessary surface functionality to prevent particle-to-particle aggregation. The collective findings from FTIR, Zeta Potential, TGA, and EDX provide a comprehensive validation of the AgNP synthesis. Together, these results demonstrate that the *Phyllanthus acidus*-mediated synthesis produces highly stable, surface-functionalized AgNPs with the structural integrity required for precise environmental sensing.

**Fig. 3:**
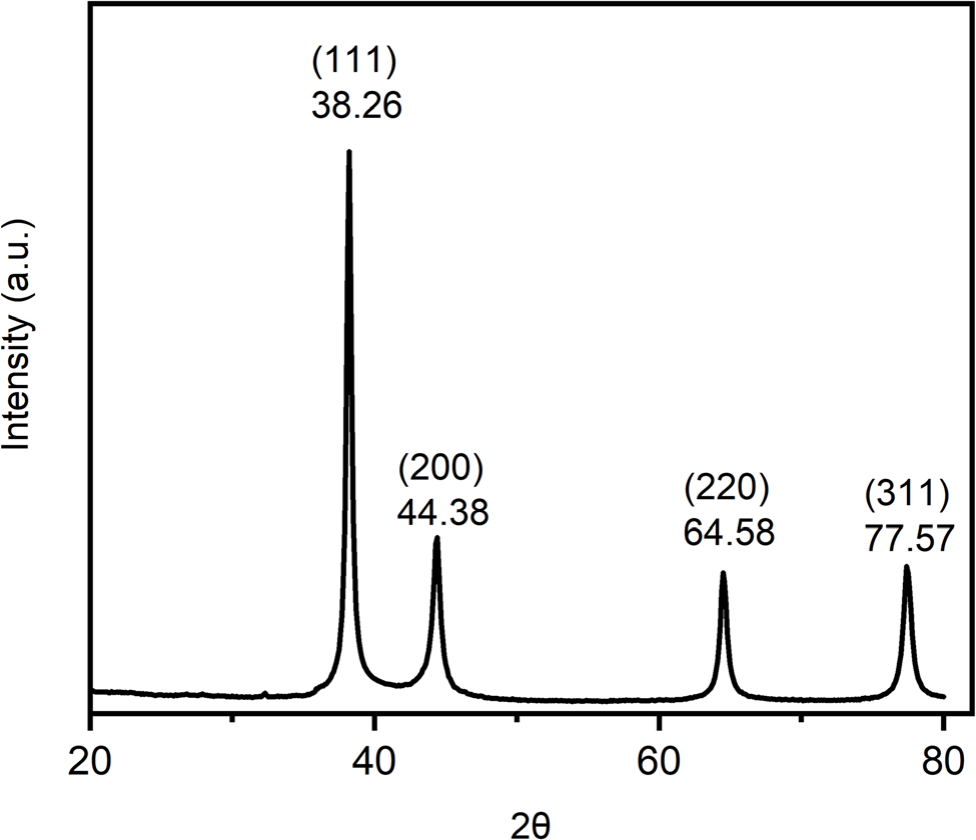
X-ray diffraction pattern of AgNPs synthesized using using *Phyllanthus acidus* leaf extract. (Condition: 1 mL of extract, 50 mL of 1 mM AgNO_3_, 60 °C, 15 minutes, and pH 10).

The crystalline nature and phase purity of the biogenically synthesized AgNPs were evaluated using X-ray diffraction (XRD) analysis. As shown in Fig. 3, the XRD pattern displays four distinct, high-intensity Bragg reflections at 2*θ* values of 38.26°, 44.38°, 64.58°, and 77.57°. These peaks correspond to the (111), (200), (220), and (311) lattice planes, respectively, which are characteristic of a face-centered cubic (fcc) silver structure (JCPDS card no. 04-0783).^14^ The predominance of the (111) reflection at 38.26° suggests that the AgNPs are highly crystalline and preferentially oriented along this plane. The interplanar *d*-spacing was calculated to be 0.221 nm, consistent with the theoretical lattice spacing for the (111) cubic structure. Using the Debye–Scherrer equation based on the Full Width at Half Maximum (FWHM) of the (111) peak (see SI). The average crystallite size was determined to be 11.76 nm. The absence of additional peaks, such as those corresponding to silver oxide (Ag_2_O) or crystalline phytochemical impurities, confirms the high phase purity of the metallic silver produced *via* the *Phyllanthus acidus* extract. These results collectively validate the formation of structurally stable and well-defined silver nanocrystals.

**Fig. 4:**
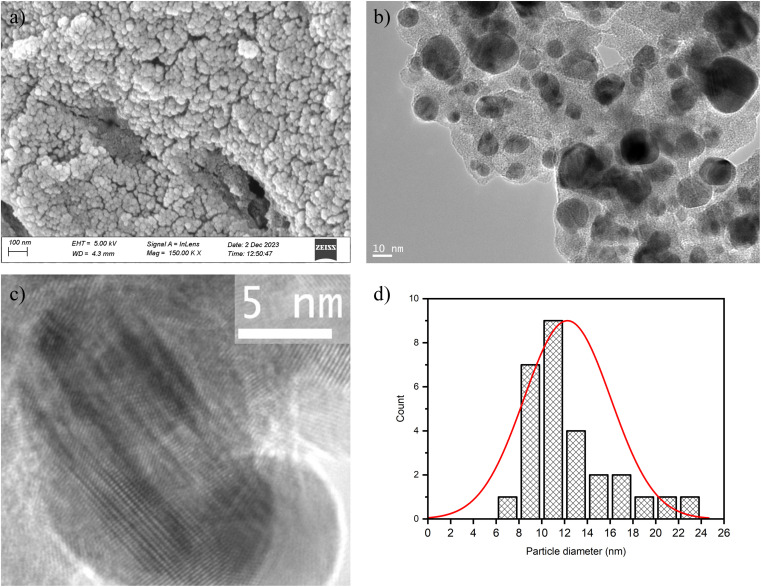
(a) SEM, (b) TEM images (c) HR_TEM and (d) size distribution plot of AgNPs synthesized using using *Phyllanthus acidus* leaf extract. (Condition: 1 mL of extract, 50 mL of 1 mM AgNO_3_, 60 °C, 15 minutes, and pH 10).

The surface morphology and internal structure of the synthesized AgNPs were characterized using Scanning Electron Microscopy (SEM) and Transmission Electron Microscopy (TEM) and HR-TEM. The SEM micrograph (Fig. 4a) reveals a predominantly granular surface morphology. The nanostructure consists of finely dispersed, spherically packed particles that exhibit a degree of interconnection. While the average particle size remains firmly within the nanometer regime, the surface displays noticeable roughness and structural defects, which are critical factors influencing the material's functional reactivity. In certain regions, the nanoparticles tend to aggregate into a continuous layer, likely driven by van der Waals forces or interparticle interactions during the drying process. While many areas show homogeneous dispersion, localized clustering is observed; this variation in uniformity is a key consideration for applications requiring consistent surface-to-volume ratios. Further insight into the particle geometry was obtained *via* TEM (Fig. 4b and c). The analysis confirms a relatively uniform distribution of spherical AgNPs (Fig. 4b), highlighting the controlled nature of the synthesis. Unlike the SEM observations which reflect surface topography the TEM images show excellent individual particle definition with minimal agglomeration, suggesting effective stabilization of the metallic cores.

### Colorimetric sensing and selectivity for Hg^2+^

**Fig. 5:**
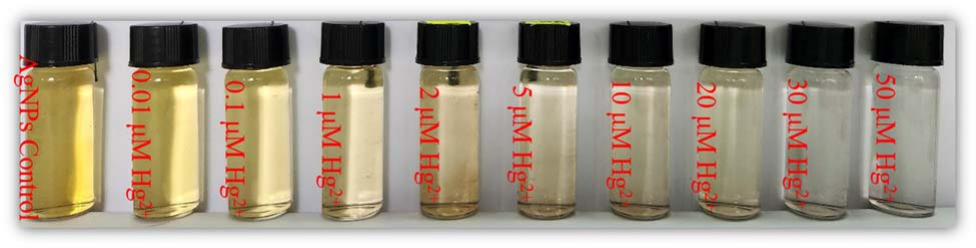
Naked-eye observation during different concentration of Hg^2+^ ion addition in AgNPs solution. A dilute solution used for better visual.

The colorimetric responses of AgNPs toward various cations were first evaluated by naked-eye (Fig. 5). Among the tested solutions, only the mixture of AgNPs with Hg^2+^ exhibited a distinct color change from brownish to colorless, while all other solutions remained unchanged (Fig. S11).^32^ This visible transformation demonstrates the potential of AgNPs as a simple and direct colorimetric indicator for Hg^2+^ detection. The results suggest that AgNPs interact with Hg^2+^ ions more selectively and specifically than alkali, alkaline earth, or transition metal ions. The difference in reduction potentials can explain this selectivity: Ag^0^ is readily oxidized to Ag^+^ by Hg^2+^, which has a higher reduction potential. As observed, the brown color of AgNPs disappears as Ag^0^ is oxidized to Ag^+^. In contrast, most alkali, alkaline earth, and transition metal ions, having lower reduction potentials than Ag^+^, are unable to oxidize Ag^0^ in AgNPs.^33^ This redox process underlies the highly selective colorimetric detection of Hg^2+^ ions using AgNPs.

### Analytical performance of the AgNP sensor for Hg^2+^ detection

**Fig. 6:**
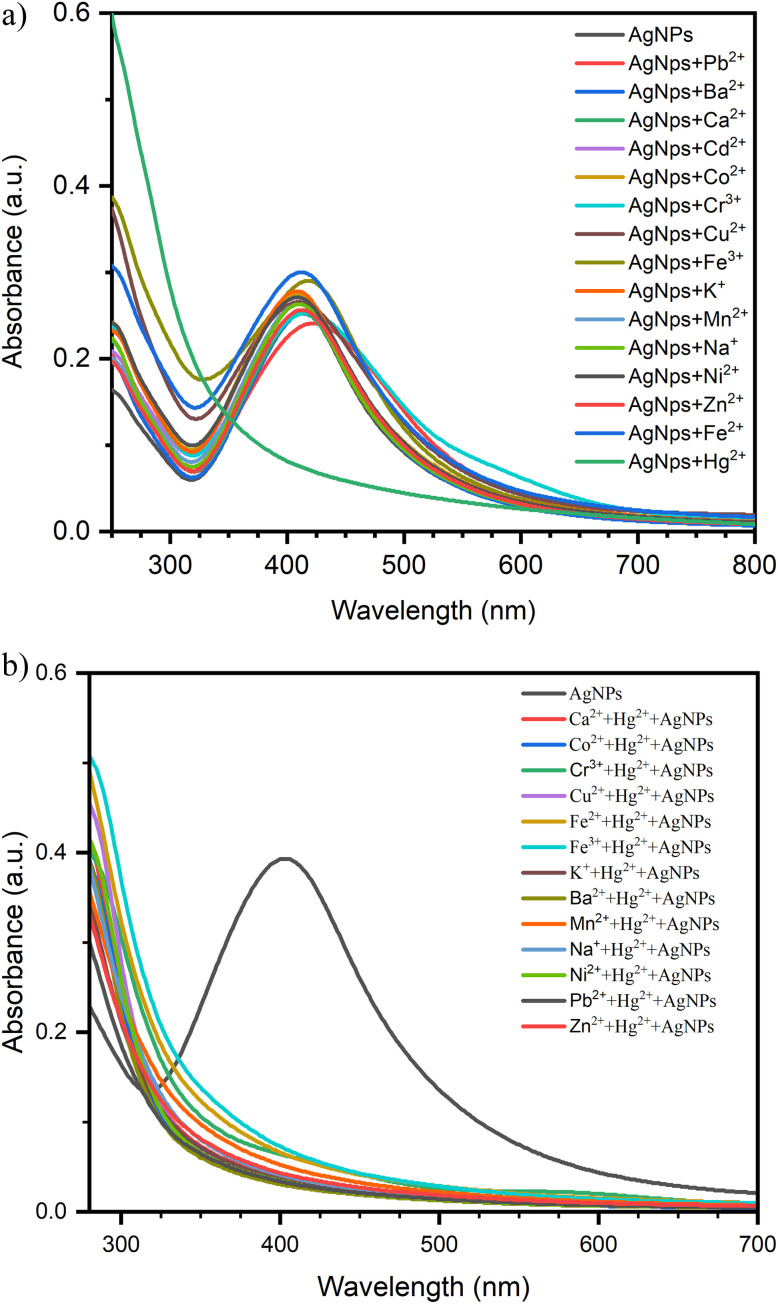
(a) SPR spectra of AgNPs solution incubated with different metal ions at the same concentration. (b) SPR spectra of AgNPs and incubated Hg^2+^ ion together with different metal ions at the same condition (right side).

The analytical performance of the AgNP sensor was rigorously evaluated based on its selectivity, sensitivity, and reliability under optimized experimental conditions. To determine the sensor's specificity, its response to Hg^2+^ was compared against a library of common metal cations, including Pb^2+^, Ba^2+^, Ca^2+^, Cd^2+^, Co^2+^, Cr^3+^, Cu^2+^, Fe^3+^, K^+^, Mn^2+^, Na^+^, Ni^2+^, Zn^2+^, and Fe^2+^. Visual inspection confirmed that only Hg^2+^ induced a transition from brown to colorless. This was quantitatively validated by measuring the Surface Plasmon Resonance (SPR) absorbance (Fig. 6a). While other cations yielded negligible spectral shifts, the AgNP–Hg^2+^ system exhibited a near-total quenching of the SPR peak, confirming high selectivity. Furthermore, competitive interference studies were conducted by introducing Hg^2+^ into solutions containing mixed interfering ions. As shown in the SPR spectra (Fig. 6b), the characteristic absorbance band of the AgNPs disappeared exclusively in the presence of Hg^2+^, regardless of the coexisting species. This demonstrates the sensor's robust anti-interference ability in complex matrices.

**Fig. 7:**
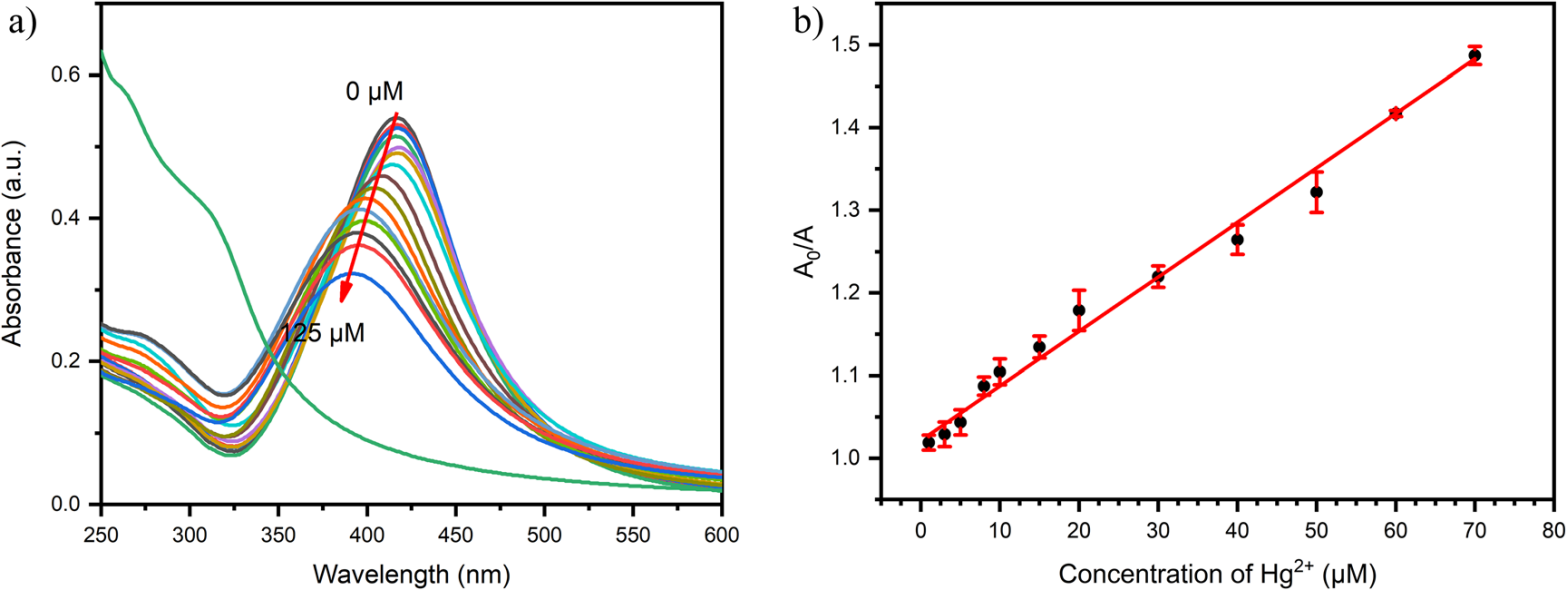
(a) UV-vis spectra of AgNPs solution with gradually increase Hg^2+^ ions in the range 0–125 µM. (b) Linear relationship between the absorbance ratio (*A*_0_/*A*) *versus* Hg^2+^ ion concentration according to figure (a).

The sensitivity of the AgNP dispersion was evaluated by monitoring the SPR response across a concentration range of 0–125 µM of Hg^2+^. As the mercury concentration increased, the absorbance at 418 nm decreased progressively (Fig. 7a), indicating the oxidative conversion of Ag^0^ to Ag^+^. To quantify this relationship, a calibration curve was constructed by plotting the absorbance ratio (*A*_0_/*A*) against the Hg^2+^ concentration. The resulting plot (Fig. 7b) displayed a strong linear correlation with a coefficient of determination (*R*^2^) of 0.9919, confirming the sensor's reliability for quantitative detection. The Limit of Detection (LOD) and Limit of Quantification (LOQ) were calculate using the following formula 3*σ*/*k* and 10*σ*/*k* respectively, where *σ* (0.00411) is the standard error of the intercept obtained from the linear regression analysis and *k* (0.00745) represents the slope of the linear calibration curve. The resulting LOD was found to be 1.65 and the LOQ was 5.5.^34^ Furthermore, the LOQ, representing the lowest concentration at which the analyte can be reliably detected with acceptable precision, was established at 5.50 µM. These values demonstrate that the AgNP dispersion serves as a highly sensitive platform for the monitoring of Hg^2+^ ions, even at low micromolar concentrations.

### Practical application and real-water sample analysis

The environmental robustness and practical utility of the AgNP-based nanoprobe were evaluated using real-world water matrices. To simulate practical detection conditions, tap water samples were sourced and spiked with known concentrations of Hg^2+^ alongside a background of common coexisting ions to assess potential matrix effects. Despite the complex chemical composition of the tap water, the AgNPs maintained high specificity for Hg^2+^, demonstrating strong anti-interference capability against domestic water additives and minerals. The analytical reliability was further validated through recovery studies, where the measured concentrations were compared against the spiked amounts. As summarized in Table 1, the recovery values fell within an acceptable analytical range, confirming that the AgNP-based colorimetric probe provides a reliable, accurate, and reproducible platform for monitoring mercury contamination in natural and municipal water systems.

**Table 1:** Real water sample analysis spiked with different amounts Hg^2+^

Water sample	Without spiked Hg^2+^	After spiked Hg^2+^ [µM]	Hg^2+^ found [µM]	Recovery [%]
Commercial drinking water	Not detected	10	9.48 ± 0.21	94.80 ± 2.10
		20	19.35 ± 0.29	96.75 ± 1.45
		30	28.07 ± 0.77	93.56 ± 2.56

### Proposed sensing mechanism: redox-driven amalgamation

**Scheme 2:**
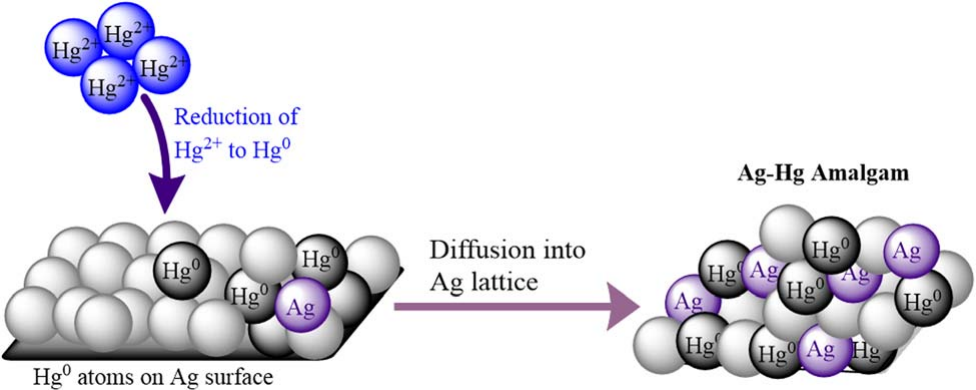
Mechanistic pathway of Ag–Hg amalgam formation during the colorimetric detection of mercury ions using AgNPs.

The selective sensing of Hg^2+^ by AgNPs is characterized by a rapid transition from a brownish dispersion to a colorless solution. This study proposes that the detection mechanism is not merely a surface interaction but a spontaneous redox-driven amalgamation process. The exceptional selectivity for mercury is rooted in the standard reduction potential of the involved species. Because Hg^2+^ (*E*^0^ ≈ +0.85 V) possesses a higher reduction potential than Ag^+^ (*E*^0^ ≈ +0.80 V), a spontaneous redox reaction occurs: 2 Ag^0^ + Hg^2+^ → 2Ag^+^ + Hg^0^.^33^ While nanoparticle aggregation often results in SPR intensity reduction, it typically does not involve the specific spectral shifts observed here. In this study, the incremental addition of Hg^2+^ resulted in a slight red shift of the SPR band. This behavior, combined with the eventual disappearance of the absorbance peak, rules out simple aggregation as the primary mechanism. Instead, the results point toward Under-Potential Deposition and the formation of an Ag–Hg amalgam (Scheme 2). Due to the high mutual solubility of silver and mercury, the reduced Hg^0^ atoms readily diffuse into the silver lattice, forming a metallic solid solution (amalgam) that lacks the characteristic SPR properties of pure AgNPs,^34^ thus rendering the solution colorless.

**Fig. 8:**
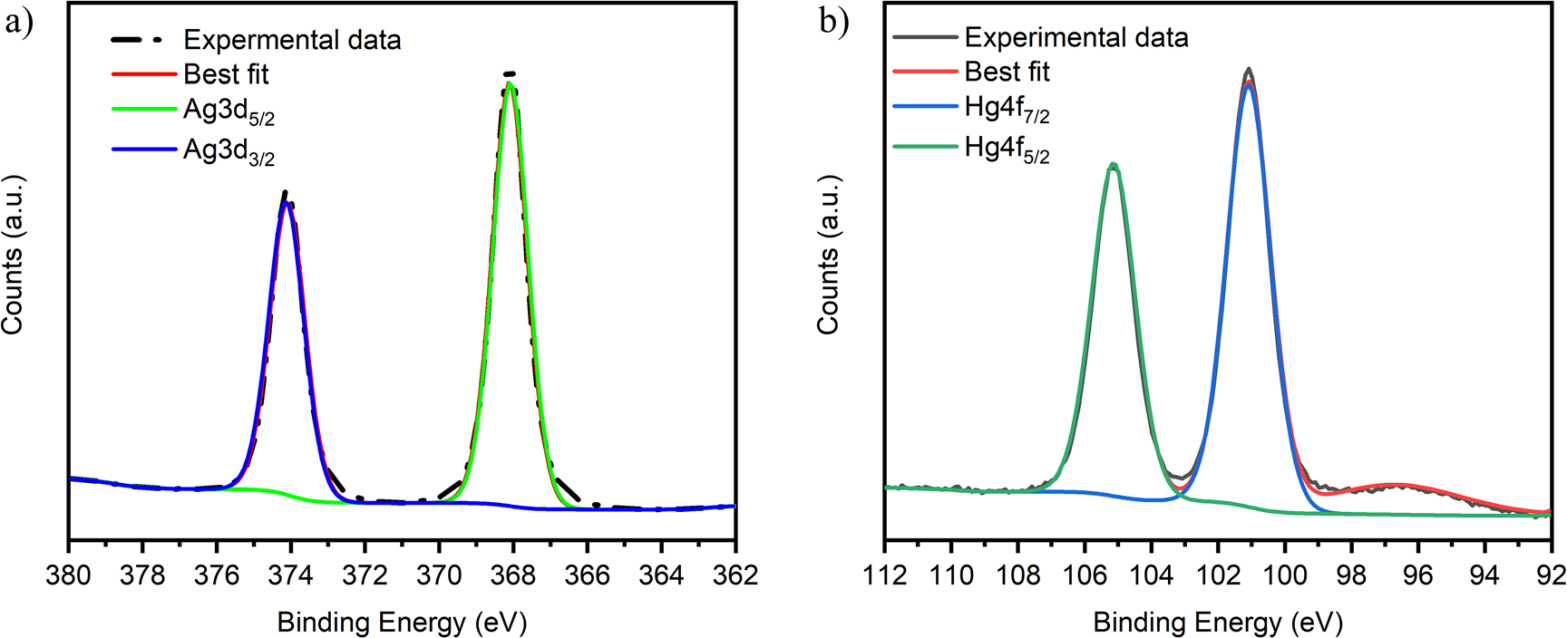
(a) SR-XPS Ag 3d core level spectra. (b) XPS spectra of Hg 4f.

To confirm the formation of the Ag–Hg alloy, Synchrotron Radiation X-ray Photoelectron Spectroscopy (SR-XPS) was employed to analyze the electronic environments of the atoms. The Ag 3d spectrum displayed two prominent peaks at 368.10 eV (3d_5/2_) and 374.16 eV (3d_3/2_), with a spin–orbit splitting of 6.06 eV, typical of metallic silver (Ag^0^) (Fig. 8a). However, a subtle shift in binding energy was observed upon Hg^2+^ exposure. This shift indicates a redistribution of electron density as mercury atoms diffuse into the silver lattice, altering the electronic structure of the silver core.^35^ The Hg 4f spectra revealed peaks at 101.06 eV (4f_7/2_) and 105.16 eV (4f_5/2_), with a separation of 4.1 eV (Fig. 8b). These binding energies are characteristic of metallic mercury (Hg^0^). Crucially, the absence of higher binding energy features, which would indicate Hg^2+^ or Hg^2+^-salts, confirms that the mercury ions were successfully reduced to their elemental state. This confirms that the silver surface acts as a reducing agent, promoting the transition of Hg^2+^ to Hg^0^ and facilitating the subsequent formation of the Ag–Hg metallic solid solution.^20,36–38^

To evaluate the analytical merit of the proposed green-synthesized AgNP sensor, its performance was compared against various previously reported silver nanoparticle-based colorimetric methods for Hg^2+^ detection. As summarized in Table 2, the proposed method exhibits a highly competitive limit of detection (LOD) and a broad linear dynamic range.

**Table 2:** Comparison with other reported AgNPs for Hg^2+^ detection method

Plant/Bio-extract	Linear range	Limit of detection (LOD)	Reference
Juglans regia (walnut)	1 × 10^−8^ to 1 × 10^−3^ M	10.65 nM	39
l-Tyrosine (sunlight irradiation)	Standard assay	16.0 nM	40
Piper chaudocanum (stem extract)	Quantitative standard	23.0 nM	41
Bistorta amplexicaulis (root extract)	1.0 to 70.0 µM	800 nM (0.8 µM)	42
Averrhoa bilimbi (fruit extract)	Standard range	1.58 µM	14
Acacia raddiana (leaf extract)	Broad assay	13.22 µM	43
Gingerol extract-	0 to 160 µM	1.6 µM	44
*Phyllanthus acidus* leaf	0 to 125 µM	1.65 µM	This work

### H_2_O_2_ detection

**Fig. 9:**
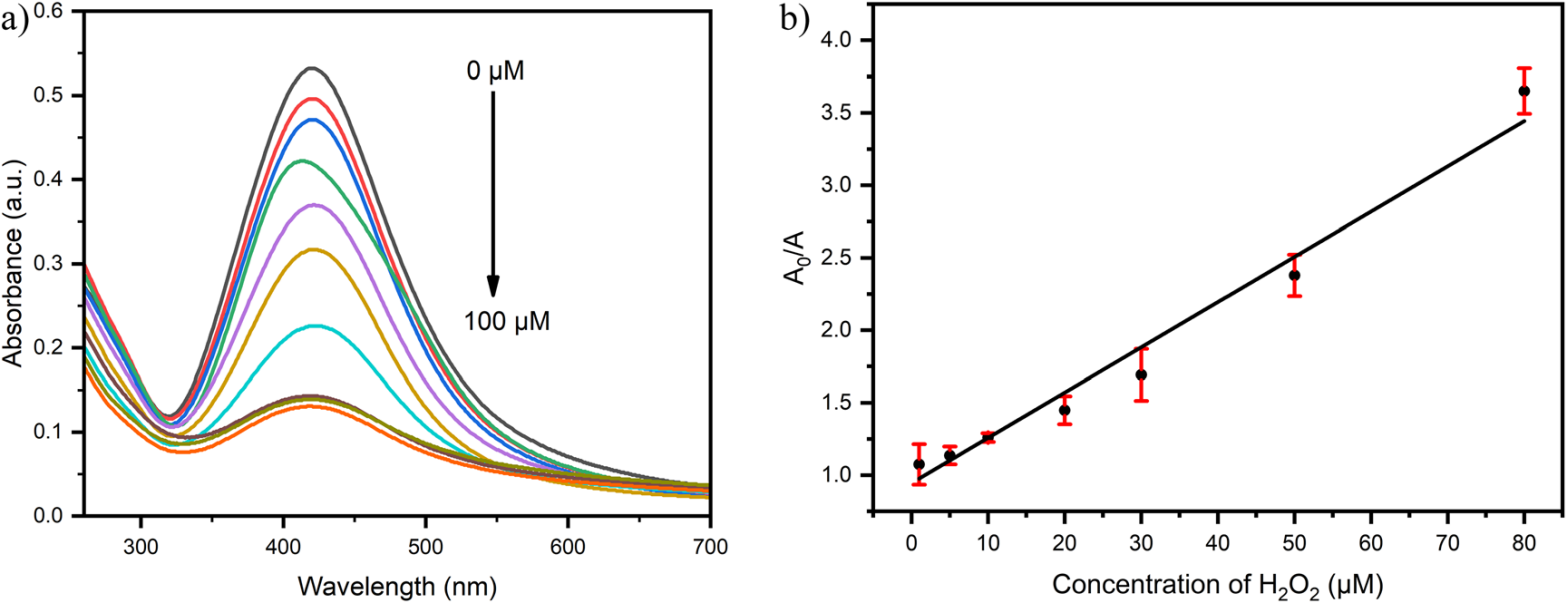
(a) UV-vis absorption spectra AgNPs solution adding various concentrations of H_2_O_2_ ions in the range 0 to 250 µM. (b) The linear relationship between the absorbance ratios (*A*/*A*_0_) *versus* H_2_O_2_ concentration.

AgNP-based colorimetric detection offers a robust and user-friendly sensing modality characterized by rapid response times and easily discernible visual readouts. In this study, synthesized AgNPs served as a visual probe for the detection of H_2_O_2_ in aqueous media. Upon the addition of H_2_O_2_ to the AgNP solution, a distinct color transition from brownish-yellow to colorless was observed, confirming the efficacy of the AgNPs as a sensing platform. This visual shift was quantitatively corroborated by UV-vis spectroscopy, where the characteristic Surface Plasmon Resonance (SPR) peak at 418 nm vanished in the presence of H_2_O_2_ (Fig. 9a). The sensing mechanism is attributed to a catalytic redox reaction potentially facilitated by the phytochemical capping agents. The addition of H_2_O_2_ generates reactive radicals that trigger the oxidative dissolution of metallic silver: Ag^0^ + H_2_O_2_ → Ag^+^ + 2OH^−^.^45^ This reaction leads to a proportional decrease in AgNP concentration as H_2_O_2_ levels rise, resulting in the attenuation of SPR intensity. Sensitivity was evaluated by incubating AgNPs with varying concentrations of H_2_O_2_ at room temperature. As illustrated in Fig. 9b, the SPR intensity decreased progressively, reaching saturation at 100 µM. A strong linear correlation (*R*^2^ = 0.9778) was established between the absorbance ratio (*A*/*A*_0_) and H_2_O_2_ concentration within the 0–100 µM range. The calculated Limit of Detection (LOD) and Limit of Quantification (LOQ) were 5.46 µM and 18.20 µM, respectively, highlighting the high sensitivity of this colorimetric probe. To evaluate the anti-interference capability of the developed system, the selectivity toward H_2_O_2_ was tested against common interfering species, including metal ions (Ca^2+^, Cu^2+^, Fe^2+^, Fe^3+^, Cr^3+^, K^+^, Ba^2+^, Mn^2+^, Ni^2+^, Zn^2+^, Na^+^, Cd^3+^ and Co^2+^) and organic molecules (Glucose, ethanol, lemon juice and uric acid). As shown in Fig. S12, the addition of a 10-fold excess of these substances induced negligible changes in the analytical response compared to the sharp, prominent signal of H_2_O_2_. This confirms that the sensor possesses exceptional selectivity for H_2_O_2_ in complex matrices.

## Experimental

### Methods and materials

Silver nitrate (AgNO_3_), mercury(ii) chloride (HgCl_2_) were purchased from Merck India. Zinc chloride, lead(ii) chloride, nickel chloride hexahydrate (NiCl_2_·6H_2_O), sodium chloride (NaCl), manganese(ii) chloride tetrahydrate (MnCl_2_·4H_2_O), potassium chloride (KCl), ferrous (ii) chloride (FeCl_2_), ferric (iii) chloride (FeCl_3_), cupric chloride dihydrate(ii) chloride (CuCl_2_·2H_2_O), chromium(iii) chloride hexahydrate (iii) (CrCl_3_·6H2O), cobalt(ii) chloride hexahydrate (CoCl_2_·6H_2_O), cadmium(ii) chloride monohydrate (CdCl_2_·H_2_O), calcium(ii) chloride dihydrate (CaCl_2_·2H_2_O), barium(ii) chloride dihydrate (BaCl_2_·2H_2_O), sodium hydroxide (NaOH) were obtained from LOBA Chemicals, India. All chemicals were of analytical grade and used without further purification. Throughout the study, deionized water was employed for all experimental procedures.

### Characterization techniques

A comprehensive characterization of the *Phyllanthus acidus* leaf extract-capped AgNPs was conducted using various analytical techniques. UV-visible absorption spectra were recorded in the range of 200–800 nm using a UV-vis spectrophotometer (Model UV-1900I, SHIMADZU, Japan). Fourier Transform Infrared (FTIR) spectroscopy was performed with an IR Spirit spectrophotometer (SHIMADZU, Tokyo, Japan), employing KBr pellets and a scan resolution of approximately 4 cm^−1^ at a controlled temperature of 25 °C. The crystallinity of the synthesized nanoparticles was examined using X-ray Diffraction (XRD) analysis on a Rigaku SmartLab system equipped with Cu-Kα radiation. Thermogravimetric Analysis (TGA) was carried out on a TGA-50 analyzer (SHIMADZU, Tokyo, Japan) under a nitrogen atmosphere with a heating rate of 10 °C min^−1^. The surface morphology and elemental composition were investigated using Scanning Electron Microscopy (SEM) and Energy Dispersive X-ray Spectroscopy (EDX) with a Hitachi SU-8000 instrument operating at accelerating voltages of 10 kV and 15 kV. Transmission Electron Microscopy (TEM) analysis was conducted using a field emission TEM (Model 2100 F, JEOL, Tokyo, Japan) to observe nanoparticle size and shape. Furthermore, Synchrotron Radiation X-ray Photoelectron Spectroscopy (SR-XPS) was employed to determine the elemental chemical states, using a K-Alpha spectrometer (ThermoFisher Scientific). Finally, the colloidal stability of the nanoparticles was assessed by measuring the zeta potential with a Zetasizer Advance system (Malvern Panalytical).

### Preparation of *Phyllanthus acidus* leaf extract

Fresh *Phyllanthus acidus* leaves were collected from Khulna University campus, Bangladesh. The leaves were thoroughly washed with deionized water to remove surface impurities, sun-dried, and subsequently ground into a fine powder. For the preparation of the aqueous extract, 10 g of the powdered leaves was mixed with 50 mL of ultra-pure water in a round-bottom flask and heated at 80 °C for 30 minutes. After cooling to room temperature, the mixture was filtered using standard filter paper to separate the liquid extract from the plant residues. The resulting extract was then centrifuged at 10 000 rpm to remove any remaining particulates, and the supernatant was collected and stored at 4 °C for subsequent experimental use.

### Green synthesis of AgNPs

The synthesis of AgNPs was initiated by mixing 1 mL of *Phyllanthus acidus* leaf aqueous extract with 50 mL of a 1 mM AgNO_3_ solution. The pH of the mixture was carefully adjusted to 10 through the gradual addition of NaOH. The reaction commenced with a colorless solution, and upon heating at 60 °C for around 15 minutes, the solution turned brown, indicating the successful formation of AgNPs. The synthesized nanoparticle suspension was then stored at 4 °C for further use.

### Visual colorimetric detection for Hg^2+^ ions using AgNPs probe

AgNPs were used as detecting probes for colorimetric Hg^2+^ sensing at room temperature. In a transparent glass vial, 1 mL of AgNPs was first added, and then 1 mL of Hg^2+^ solution with varying concentrations was added. Lastly, the color shift in the AgNPs probe was visible to the unaided eye.

### Evaluating the performance of the AgNPs sensor for Hg^2+^ detection

Stock solutions (1.0 mM) of various metal ions, including Zn^2+^, Pb^2+^, Ni^2+^, Na^+^, Mn^2+^, K^+^, Fe^3+^, Fe^2+^, Cu^2+^, Cr^3+^, Co^2+^, Cd^2+^, Ca^2+^, Ba^2+^, and Hg^2+^ were prepared by dissolving the corresponding metal salts in deionized water. To evaluate the selectivity of the synthesized AgNPs toward Hg^2+^ ions, individual metal ion solutions were added separately to the AgNP suspension, and any visible color change was noted. The corresponding UV-visible absorption spectra were recorded using a UV-vis spectrophotometer after each metal ion addition. Subsequently, Hg^2+^ ion detection was performed both in the presence and absence of other competing metal ions. In another set of experiments, all metal ions, including Hg^2+^, were added simultaneously to the AgNP solution in equal concentrations to observe potential interference effects. Furthermore, to determine the sensitivity of the detection method, a series of Hg^2+^ solutions with concentrations ranging from 0 to 120 µM were incrementally introduced into the AgNP solution under optimized conditions, and the absorbance was recorded accordingly.

### Application of nanoprobe for real water sample analysis

The real water sample, commercial drinking water, was tampered with 10, 20, 30, 40, and 50 µM of Hg^2+^ solution to determine the Hg^2+^ concentration and recovery percentages. The known spiked samples of 10, 20, 30, 40, and 50 µM of Hg^2+^ solution were employed in the conventional addition procedure. The goal of the detection procedure was to create a standard curve for calculating the Hg^2+^ ion in unidentified samples.

### H_2_O_2_ detection

H_2_O_2_ colorimetric detection was investigated using a synthesized AgNPs probe for visual detection in water using the naked eye at room temperature. First, 1 mL of AgNPs was added to a clear glass vial, followed by the addition of 1 mL of different concentrations of H_2_O_2_ solution, and the observed color change of the solution mixture was with the naked eye. The sensitivity of the AgNPs probe for H_2_O_2_ detection was conducted by adding a series of solutions with varying concentrations of H_2_O_2_ solution from 0 to 100 µM to the AgNPs solutions under optimal experimental conditions, and the absorbance was recorded successively.

## Conclusions

In this work, a green-synthesized AgNP-based colorimetric probe was successfully developed and implemented for the sensitive detection of Hg^2+^ ions and H_2_O_2_. The probe demonstrated rapid response kinetics, characterized by a distinct “naked-eye” color transition from brownish-yellow to colorless. This optical shift showed a strong linear dependence on analyte concentration. For Hg^2+^ linear range of 0–70 µM was established (*R*^2^ = 0.9919) with LOD of 1.65 µM and a LOQ of 5.50 µM. Similarly, H_2_O_2_ detection exhibited a linear range of 0–100 µM (*R*^2^ = 0.9778), yielding an LOD of 5.46 µM and an LOQ 18.20 µM. The probe exhibited exceptional selectivity for Hg^2+^ against a broad spectrum of competing metal ions, including Zn^2+^, Pb^2+^, Ni^2+^, Na^+^, Mn^2+^, K^+^, Fe^3+^, Fe^2+^, Cu^2+^, Cr^3+^, Co^2+^, Cd^2+^, Ca^2+^, and Ba^2+^. The practical utility of the sensor was further validated through the successful quantification of Hg^2+^ in real water samples with high recovery rates. Mechanistically, SR-XPS analysis confirmed the interaction between the AgNPs and Hg^2+^ ions, revealing a simultaneous partial reduction of Hg^2+^ to Hg^0^ and the oxidation of Ag^0^ to Ag^+^. These results suggest the formation of a mixed AgNP–Ag/Hg amalgam system.

In conclusion, this synthesized AgNP probe provides an eco-friendly, cost-effective, and straightforward platform for monitoring Hg^2+^ and H_2_O_2_ in aqueous environments. By facilitating the early identification of heavy metal pollutants, this research supports efforts to mitigate public health hazards and environmental risks associated with waterborne contaminants.

## Author contributions

Md. Ahad Mahamud Nahim: methodology, formal analysis, investigation, data curation, validation. Md. Taufiqul Islam: validation, methodology, formal analysis. Saurav Kumar Das: validation, methodology, data curation. A.B.M. Nazmul Islam: writing – original draft, conceptualization, investigation. Rumpa Kundu: visualization, validation, formal analysis. Md. Abu Rayhan Khan: visualization, validation, methodology. Shofiur Rahman: writing – review and editing, writing – original draft, validation, supervision, conceptualization. Mahmoud A. Al-gawati: writing – review and editing, validation, conceptualization. Md. Ahsan Habib: writing – original draft, validation, supervision, software, investigation, funding acquisition, conceptualization.

## Conflicts of interest

There are no conflicts to declare.

## Supplementary Material

RA-016-D6RA02784A-s001

## Data Availability

Supplementary information (SI): additional characterization spectra and figures, tables, and supporting data related to the results presented in this article. See DOI: https://doi.org/10.1039/d6ra02784a.
