# Supplementary material for: Eco-friendly silver nanoparticle-based dual sensors for environmental toxicants: Hg2+ and H2O2
Source: RSC Adv. 2026 Jul 16. Online ahead of print. doi: 10.1039/d6ra02784a (PMC13373593; doi:10.1039/d6ra02784a)
Supplement: RA-OLF-D6RA02784A-s001 [file RA-OLF-D6RA02784A-s001.pdf]

Supplementary Information (SI) for RSC Advances.
This journal is © The Royal Society of Chemistry 2026

Supporting Information

Eco-Friendly Silver Nanoparticle-Based Dual Sensors for Environmental Toxicants: Hg^{2+} and H_2O_2

Md. Ahad Mahamud Nahim,[a] Md Toufiqul Islam,[a] Saurav Kumar Das,[a] A.B.M. Nazmul Islam,[a] Rumpa Kundu,[b] Md. Abu Rayhan Khan,[c] Shofiur Rahman,*[d] Mahmoud A. Al-gawati,[d] and Md. Ahsan Habib,*[a]

[a] Chemistry Discipline, Khulna University, 3rd academic building, Khulna University, Khulna-9208, Bangladesh.
[b] Graduate School of Environmental Studies, Tohoku University, 6−6−20 Aoba, Aramaki, Aoba−ku, Sendai 980-8579, Japan.
[c] Department of Chemistry, Mississippi State University, USA
[d] Biological and Environmental Sensing Research Unit, King Abdullah Institute for Nanotechnology, King Saud University, Riyadh 11451, Saudi Arabia

Corresponding authors: (ahsanhru@chem.ku.ac.bd and, mrahman1@ksu.edu.sa).
†Present address: Chemistry Discipline, Khulna University, 3rd academic building, Khulna University, Khulna-9208, Bangladesh and Biological
and Environmental Sensing Research Unit, King Abdullah Institute for Nanotechnology, King Saud University, Riyadh 11451, Saudi Arabia

Contents

Sl. No.	Description	Page no.
S1	Figure S1. UV-Vis spectra of AgNPs at different temperature. Condition: AgNO3: 50 mL of 1 mM; Phyllanthus acidus leaf extract: 1 mL; time: 15 minutes and pH: 10.	3
S2	Figure S2. UV-Vis spectra of AgNPs at different time. Condition: AgNO3: 50 mL of 1 mM; Phyllanthus acidus leaf extract: 1 mL; temperature: 60 °C; minutes and pH: 10.	3
S3	Figure S3. UV-Vis spectra of AgNPs using different amount of Phyllanthus acidus leaf extract. Condition: AgNO3: 50 mL of 1 mM; temperature: 60 °C; time: 15 minutes and pH: 10.	4
S4	Figure S4. UV-Vis spectra of AgNPs using different concentration of AgNO3 with volume 50 mL. Condition: Phyllanthus acidus leaf extract: 1 mL; Temperature: 60 °C; time: 15 minutes and pH: 10.	4
S5	Figure S5. UV-Vis spectra of AgNPs at different pH. Condition: AgNO3: 50 mL of 1 mM; Phyllanthus acidus leaf extract: 1 mL; Temperature: 60 °C; time: 15 minutes.	5
S6	Figure S6. Zeta potential plot for AgNPs. Condition: 1 mL of extract, 50 mL of 1 mM AgNO3, 60 °C, 15 minutes, and pH 10.	5
S7	Figure S7. UV-Vis spectra of AgNPs for long-term storage stability stored at 4°C over 9 months. Synthesis condition: 1 mL of extract, 50 mL of 1 mM AgNO3, 60 °C, 15 minutes, and pH 10.	6
S8	Figure S8. TGA graph for AgNPs recorded at a heating rate of 10 °C/min under a nitrogen atmosphere. Condition: 1 mL of extract, 50 mL of 1 mM AgNO3, 60 °C, 15 minutes, and pH 10.	7
S9	Figure S9. EDX spectrum of AgNPs synthesized using Phyllanthus acidus leaf extract. (Condition: 1 mL of extract, 50 mL of 1 mM AgNO3, 60 °C, 15 minutes, and pH 10.	7
S 10	Figure S 10. SR-XPS survey spectra of AgNPs-Hg	8
S11	Figure S 11. Colorimetric selectivity profile of AgNPs toward Hg^{2+} ions compare with various competing metal ions.	8
S1	Table S1: Crystalline parameter for synthesized AgNPs from powder xrd data	~~8~~ 9
S12	Figure S12. a) SPR spectra of AgNPs solution incubated with different organic molecules. b) SPR spectra of AgNPs solution incubated H_2O_2 together with different organic molecules at the same condition. c) SPR spectra of AgNPs and incubated H_2O_2 together with different metal ions at the same conditions.	10

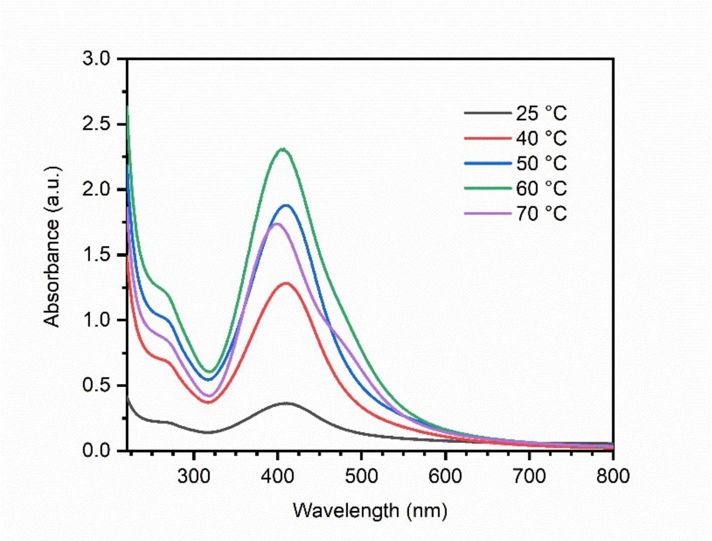

Figure S1. UV-Vis spectra of AgNPs at different temperature. Condition: $AgNO_3$: 50 mL of 1 mM; *Phyllanthus acidus* leaf extract: 1 mL; time: 15 minutes and pH: 10.

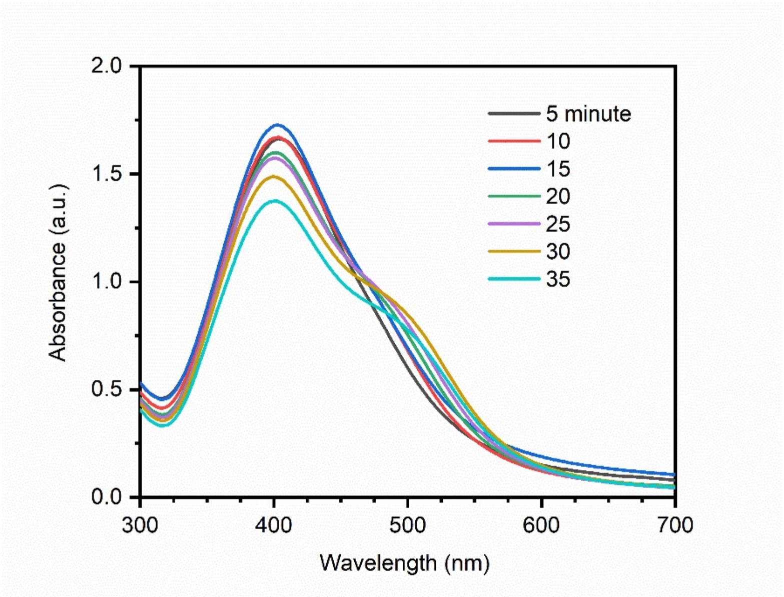

Figure S2. UV-Vis spectra of AgNPs at different time. Condition: $AgNO_3$: 50 mL of 1 mM; *Phyllanthus acidus* leaf extract: 1 mL; temperature: 60 °C; minutes and pH: 10.

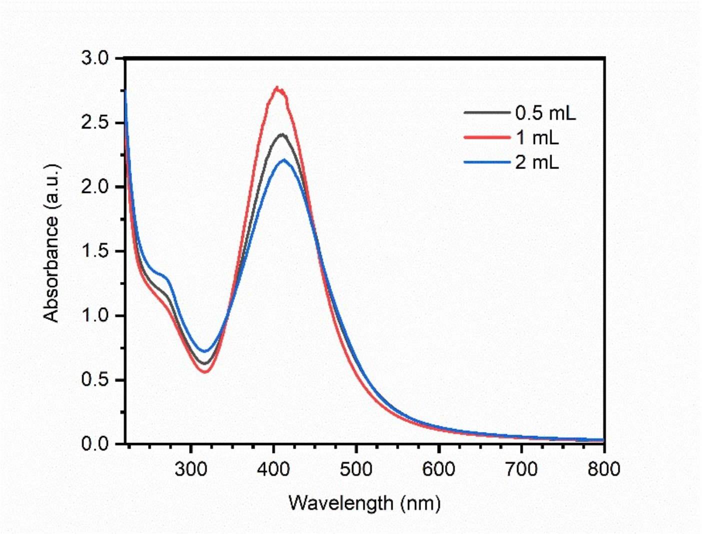

Figure S3. UV-Vis spectra of AgNPs using different amount of *Phyllanthus acidus* leaf extract. Condition: $AgNO_3$: 50 mL of 1 mM; temperature: 60 °C; time: 15 minutes and pH: 10.

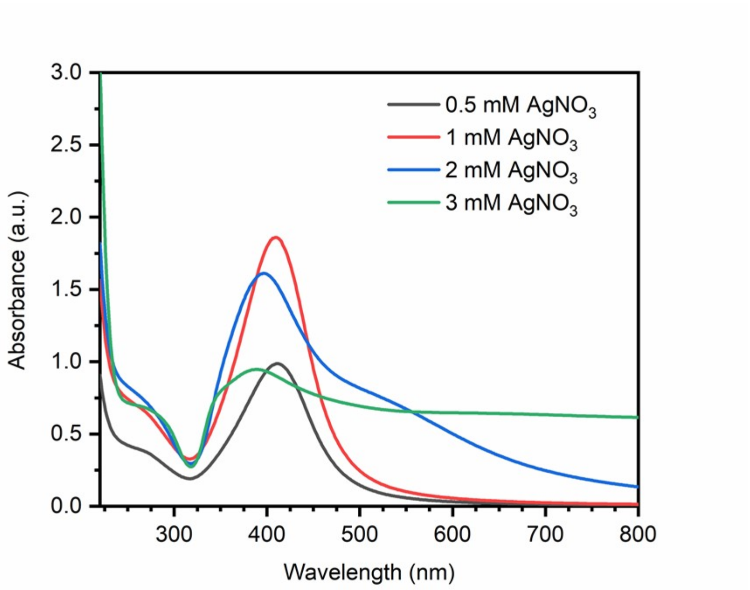

Figure S4. UV-Vis spectra of AgNPs using different concentration of $AgNO_3$ with volume 50 mL. Condition: *Phyllanthus acidus* leaf extract: 1 mL; Temperature: 60 °C; time: 15 minutes and pH: 10.

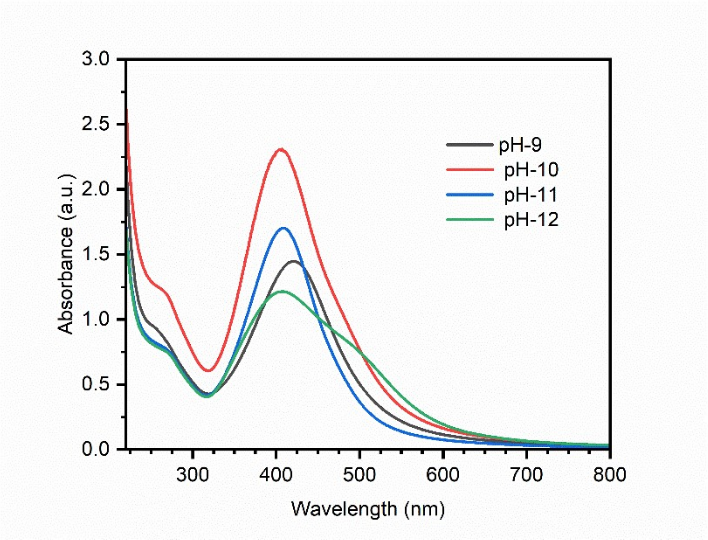

Figure S5. UV-Vis spectra of AgNPs at different pH. Condition: $AgNO_3$: 50 mL of 1 mM; *Phyllanthus acidus* leaf extract: 1 mL; Temperature: 60 °C; time: 15 minutes.

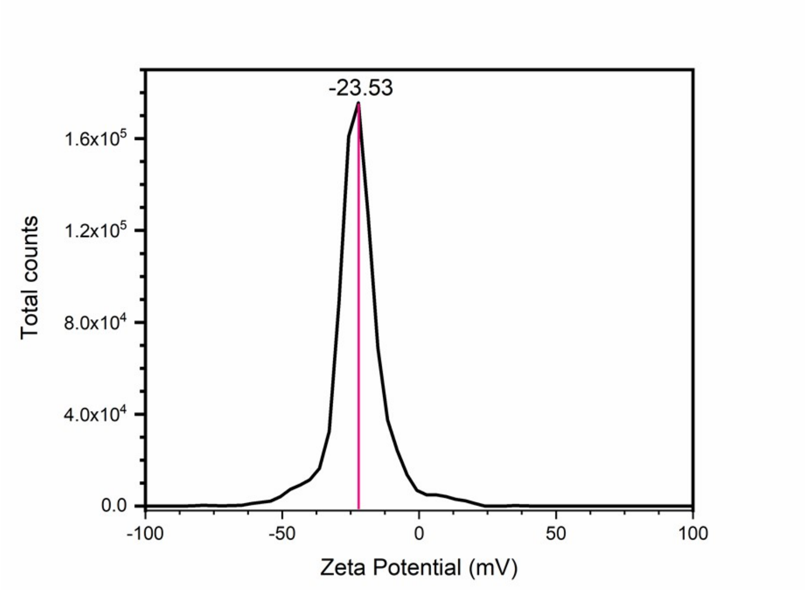

Figure S6. Zeta potential plot for AgNPs. Condition: 1 mL of extract, 50 mL of 1 mM AgNO3, 60 °C, 15 minutes, and pH 10.

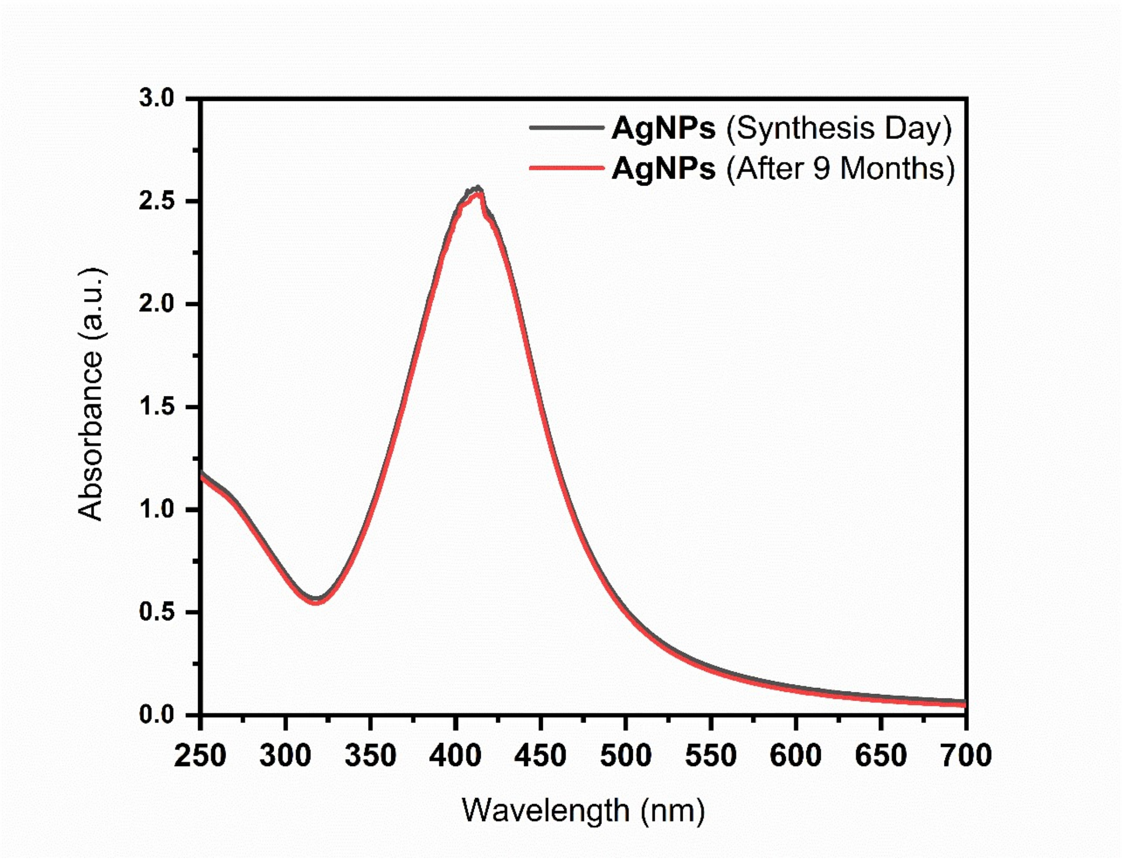

Figure S7. UV-Vis spectra of AgNPs for long-term storage stability stored at 4°C over 9 months. Synthesis condition: 1 mL of extract, 50 mL of 1 mM AgNO3, 60 °C, 15 minutes, and pH 10.

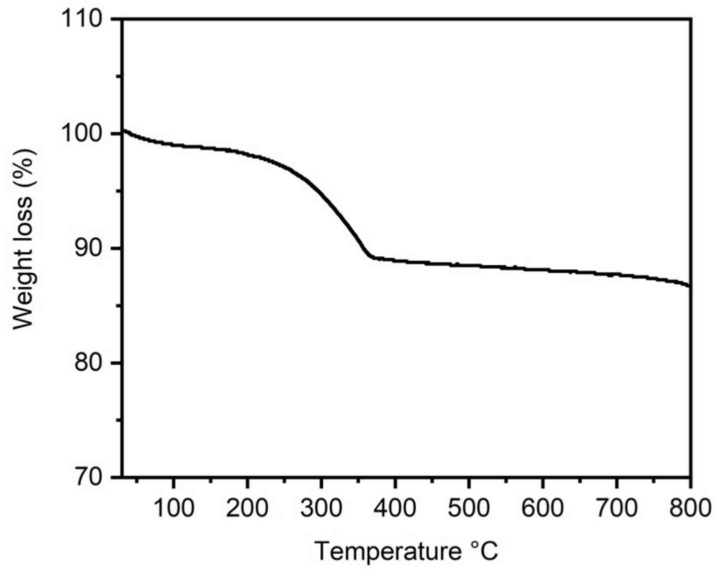

Figure S 8. TGA graph for AgNPs recorded at a heating rate of 10 °C/min under a nitrogen atmosphere. Condition: 1 mL of extract, 50 mL of 1 mM $AgNO_3$, 60 °C, 15 minutes, and pH 10.

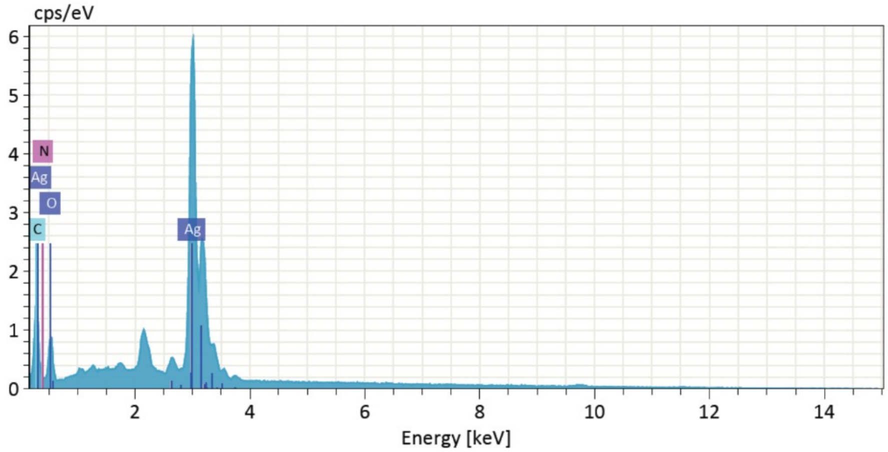

Figure S 9. EDX spectrum of AgNPs synthesized using *Phyllanthus acidus* leaf extract. (Condition: 1 mL of extract, 50 mL of 1 mM $AgNO_3$, 60 °C, 15 minutes, and pH 10.

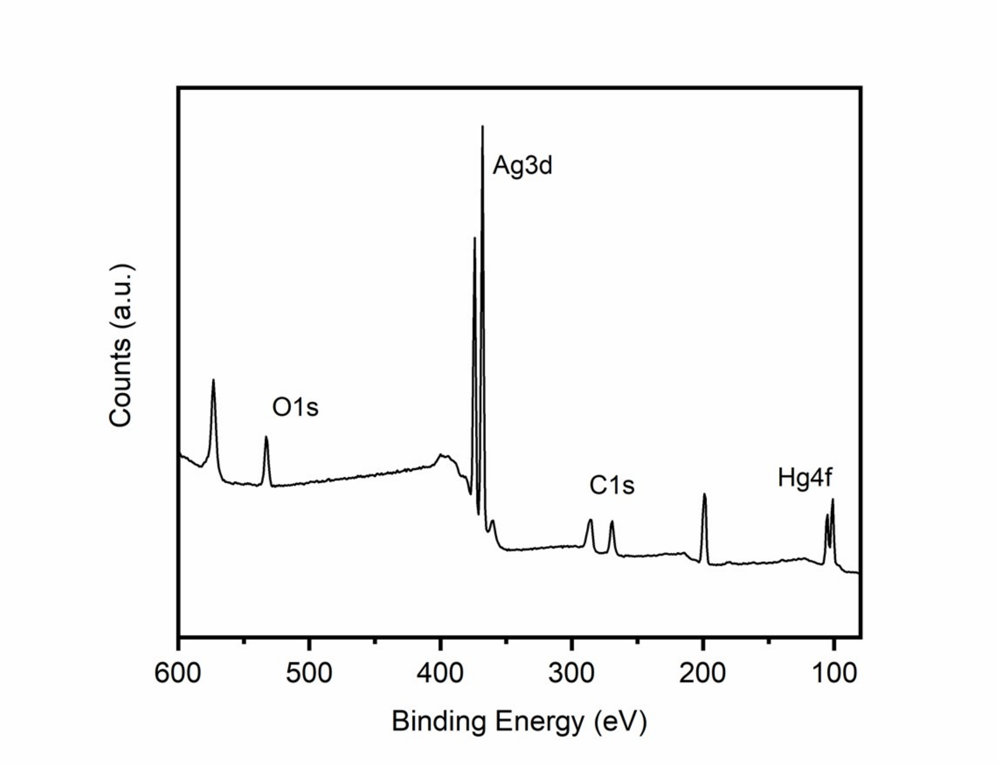

Figure S10. SR-XPS survey spectra of AgNPs-Hg

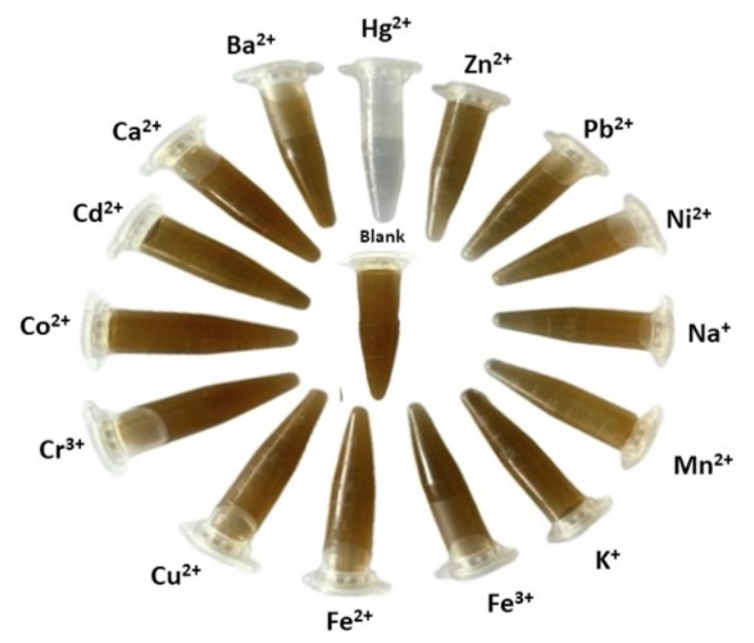

Figure S 11. Colorimetric selectivity profile of AgNPs toward Hg^{2+} ions compare with various competing metal ions.

Crystalline size calculation from XRD data:

Crystalline size was calculated using the Debye–Scherrer equation.

$$D = \frac{K\lambda}{\beta cos\theta} \qquad (1)$$

Where,

D = mean crystalline size (nm)

K = shape factor (commonly 0.9 for roughly spherical crystallites; use 0.89–1.0 depending on shape)

λ = X-ray wavelength (e.g. Cu Kα\alphaα = 1.5406 Å or 0.15406 nm

β = FWHM of the diffraction peak corrected for instrumental broadening, expressed in radians (measured in 2θ units and converted to radians).

θ = Bragg angle = (2θ peak position)/2, in radians.

Using the following equation and value, calculate the crystalline size of AgNPs, and the average crystalline size found to be 11.56 nm

(hkl)	2θ (Bragg's diffraction angle)	β (full width at half-maximum (FWHM) (radians)	x-ray wavelength (nm)	K	D (crystalline size (nm))
(111)	38.26	0.010690	0.15406	0.9	13.51
(200)	44.38	0.014108	0.15406	0.9	10.61
(220)	64.58	0.013484	0.15406	0.9	12.16
(311)	77.57	0.016494	0.15406	0.9	10.78
		**Average crystalline size (nm)**			11.76

Table S1: Crystalline parameter for synthesized AgNPs from powder XRD data

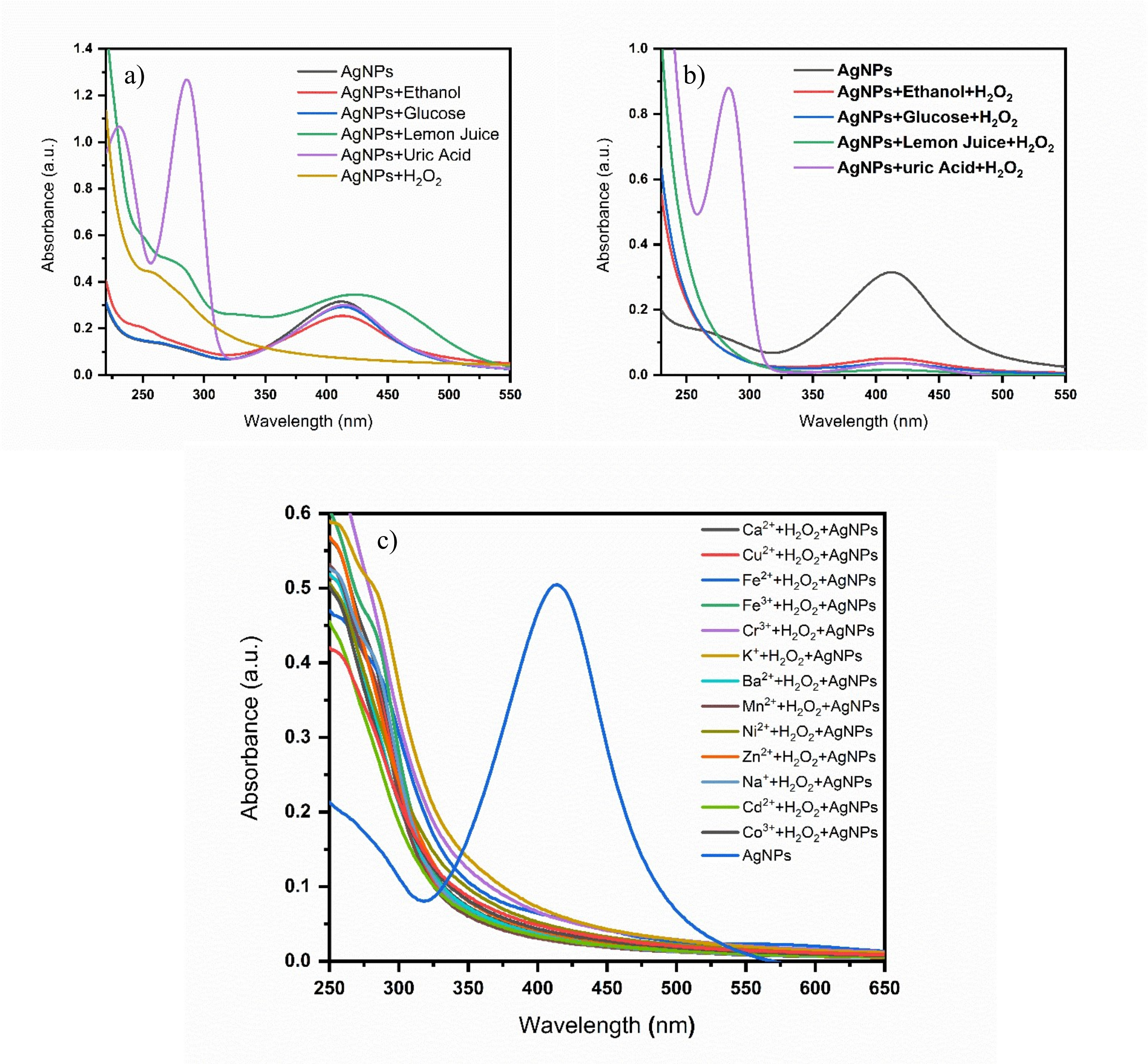

Figure S12. a) SPR spectra of AgNPs solution incubated with different organic molecules. b) SPR spectra of AgNPs solution incubated H_2O_2 together with different organic molecules at the same condition. c) SPR spectra of AgNPs and incubated H_2O_2 together with different metal ions at the same condition
